# The dynamic trophic niche of an island bird of prey

**DOI:** 10.1002/ece3.6856

**Published:** 2020-10-03

**Authors:** Ulises Balza, Nicolás A. Lois, Michael J. Polito, Klemens Pütz, Amira Salom, Andrea Raya Rey

**Affiliations:** ^1^ Laboratorio de Ecología y Conservación de Vida Silvestre Centro Austral de Investigaciones Científicas (CADIC‐CONICET) Ushuaia Argentina; ^2^ Departamento de Ecología, Genética y Evolución Facultad de Ciencias Exactas y Naturales Universidad de Buenos Aires Buenos Aires Argentina; ^3^ Instituto de Ecología Genética y Evolución de Buenos Aires CONICET Buenos Aires Argentina; ^4^ Department of Oceanography and Coastal Sciences Louisiana State University Baton Rouge LA USA; ^5^ Antarctic Research Trust Bremervörde Germany; ^6^ Instituto de Ciencias Polares, Ambiente y Recursos Naturales (ICPA) Universidad Nacional de Tierra del Fuego (UNTdF) Ushuaia Argentina; ^7^ Wildlife Conservation Society Representación Argentina Buenos Aires Argentina

**Keywords:** ecological specialization, floaters, individual ecology, intraspecific variation, nicheROVER, pulsed resources, SIBER, SIDER, trophic ecology

## Abstract

Optimal foraging theory predicts an inverse relationship between the availability of preferred prey and niche width in animals. Moreover, when individuals within a population have identical prey preferences and preferred prey is scarce, a nested pattern of trophic niche is expected if opportunistic and selective individuals can be identified. Here, we examined intraspecific variation in the trophic niche of a resident population of striated caracara (*Phalcoboenus australis*) on Isla de los Estados (Staten Island), Argentina, using pellet and stable isotope analyses. While this raptor specializes on seabird prey, we assessed this population's potential to forage on terrestrial prey, especially invasive herbivores as carrion, when seabirds are less accessible. We found that the isotopic niche of this species varies with season, age, breeding status, and, to a lesser extent, year. Our results were in general consistent with classic predictions of the optimal foraging theory, but we also explore other possible explanations for the observed pattern. Isotopic niche was broader for groups identified a priori as opportunistic (i.e., nonbreeding adults during the breeding season and the whole population during the nonbreeding season) than it was for individuals identified a priori as selective. Results suggested that terrestrial input was relatively low, and invasive mammals accounted for no more than 5% of the input. The seasonal pulse of rockhopper penguins likely interacts with caracara's reproductive status by constraining the spatial scale on which individuals forage. Niche expansion in spatially flexible individuals did not reflect an increase in terrestrial prey input; rather, it may be driven by a greater variation in the types of marine prey items consumed.

## Graphical abstract

We studied intraspecific variation in the trophic niche of a resident population of striated caracara on Isla de los Estados, Argentina, using pellet and stable isotope analyses. Isotopic niche of this species varies with season, age, breeding status, and, to a lesser extent, year. Our results were in general consistent with classic predictions of the optimal foraging theory. The seasonal pulse of rockhopper penguins likely interacts with the reproductive status of caracaras by constraining the spatial scale on which the individuals forage. The expansion of niche in spatially flexible individuals does not simply reflect an increase in terrestrial prey input; rather, it may be driven by a greater variation in the types of marine prey items consumed.

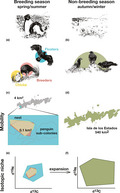

## 1 INTRODUCTION

Variability in individual diets can have strong effects on community structure (Des Roches et al., 2018) and supports population persistence (Ducatez et al., 2020). Intraspecific variation in diet can be related to several factors, such as sex dimorphism (e.g., Ebenman & Nilsson, 1982), morphology (e.g., Snowberg et al., 2015), life history stages (e.g., Pratte et al., 2018), food web sources (e.g., Tarroux et al., 2012), population density (e.g., Svanbäck & Persson, 2004), habitat preferences (e.g., Quevedo et al., 2009), association with human subsidies (e.g., Newsome et al., 2015), and landscape heterogeneity (e.g., Darimont et al., 2009), among others. Optimal foraging theory (OFT) is one of the principal concepts derived from optimization theory and sustains multiple hypotheses regarding how populations are shaped by natural selection (MacArthur & Pianka, 1966). One of the main predictions of OFT is that variation in prey choices is driven by intraspecific competition and produces an inverse relationship between preferred resource availability and trophic niche width (Araújo et al., 2008; Pyke, 1984). The predictions derived from OFT have been supported by past studies examining relatively simple model systems, such as central‐place foraging (Perry & Pianka, 1997).

An expansion of OFT includes predictions of how individuals behave in relation to their own rank of preferences. In both the Shared Preferences model and the Competitive Refuge model, all individuals share their first‐ranked preferred prey. However, while in the Shared Preferences model they also share the ranking of preferences for all possible prey, in the Competitive Refuge model the lower‐value prey type rank differs among individuals (Svanbäck & Bolnick, 2005). The different predictions for these models can be tested using individual‐resource networks (Pires et al., 2011). In the Shared Preferences model, a nested pattern is expected, with “selective” individuals (i.e., those who succeed in their first‐ranked prey consumption) being a subset of “opportunistic” ones.

Birds of prey represent promising model species to study variation in individual diet specialization. In these species, foraging and nesting is associated with hierarchical and agonistic interactions among individuals, which allows a classification between opportunistic and selective individuals (Newton, 1979). During the breeding season, chicks require high frequency and high‐quality food deliveries, spatially restricting breeding individuals to an area around the nest and enforcing a highly selective prey choice (Newton, 1979). In contrast, nonbreeding adults (hereafter “floaters,” following Smith, 1978) have relatively loose ranges, with less access to valuable resources and less selectivity in prey choices. However, floaters are attracted to occupied territories, both for food and for chances of future territorial acquisition (Ferrer et al., 2015; Newton, 1991). Moreover, many raptors are plastic foragers known to exploit pulsed resources in terrestrial systems (e.g., Therrien et al., 2014) and to rapidly incorporate novel invasive mammals in their diet (e.g., Barbar et al., 2016).

**FIGURE 1:**
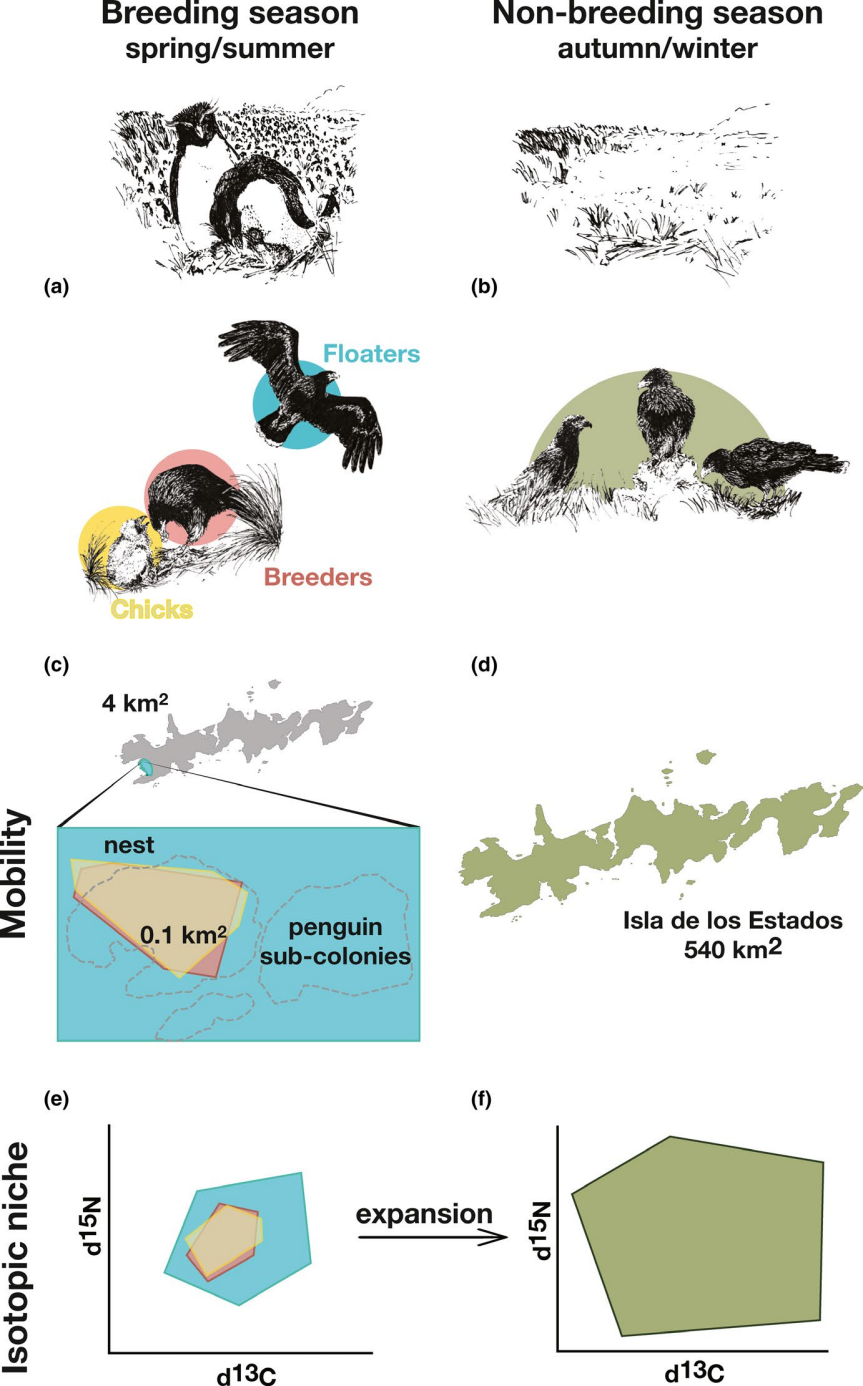
Hypothesis framework for this study, color‐coded as follows: floaters in light blue, breeders in pink, chicks in yellow, and all age classes during nonbreeding season in green. During the breeding season (a), we predict mobility for breeders and chicks will be spatially restricted to an area around the nest, while floaters, though also attracted to penguin colonies, will maintain a larger spatial range, which includes, at least, the 4 km^2^ area of Franklin Bay (c). We thus expect breeders' and chicks' isotopic niches to be a subset of the floaters' niche (e). During the nonbreeding season (b), while the rockhopper penguins overwinter at sea, we propose a range expansion for all age classes (d) with a predicted incorporation of new prey sources and a consequent isotopic niche expansion (f)

The striated caracara (*Phalcoboenus australis*, hereafter “caracara”) is a near threatened bird of prey restricted to islands in southern South America (BirdLife International, 2018; Frere et al., 1999; Marín et al., 2006; Reeves et al., 2018). During the breeding season, they associate with seabird colonies, breeding in their proximity and feeding on eggs, chicks, adults, and carcasses (Catry et al., 2008; Liljesthröm et al., 2008; Strange, 1996). It is expected that, to ensure breeding success, nesting attempts are preferentially associated with (i.e., restricted to) foraging habitats that include seabird nesting patches (Balza et al., 2017; Catry et al., 2008; Strange, 1996). Floaters, although also attracted to the colonies, are likely to be excluded, or have limited access to them due to territorial behavior of breeding pairs (Figure 1). This suggest that floaters might use a wider range of resources than breeding adults and chicks given their lower (if any) mobility restriction, together with their relative restricted access to seabird colonies. During the nonbreeding season, when seabirds have deserted their breeding grounds to winter in the open sea, caracaras shift their feeding habits. Although they might still feed on marine prey, such as intertidal organisms and nonmigrant but less abundant populations of seabirds and marine mammals (Strange, 1996), some shift to feeding on terrestrial prey items (e.g., geese *Chloephaga* sp., terrestrial invertebrates, and livestock, Harrington et al., 2018; Rexer‐Huber & Bildstein, 2013). However, no study on the feeding ecology of the species is available for the populations in the Fuegian archipelago, where interactions between food resources are thought to be different (see Section 2.1, Study area).

The aim of this study was to investigate intraspecific variation in the trophic niche of caracaras on a resident population in Isla de los Estados (southern Argentina), a continental island with no human population apart from an Argentine Army station manned by four marines. We specifically tested for the Shared Preferences model, under which a nested pattern is expected, with selective individuals' niche being a subset of opportunists' niche, when the preferred prey is scarce (Pires et al., 2011). We believe this could be the case in our study system as seasonally migratory southern rockhopper penguins (*Eudyptes chrysocome*) appear to be the dominant prey choice, while all other prey types are less clustered and abundant (see Materials and methods); thus, no selectivity is expected apart from the one related to rockhoppers (Figure 1).

We tested for differences in trophic niche width and overlap relative to age, breeding status, and seasonal changes in resource availability using pellet and stable isotope analyses. We also use stable isotope mixing models to explore whether the population, especially opportunistic and spatially flexible individuals (i.e., floaters during the breeding season and the entire population in the nonbreeding season), could be surrogating their lack of access to seabird colonies by feeding on terrestrial resources. Particularly, we focus on the contribution of carrion of invasive herbivores as a subsidy, which could have important management consequences for the conservation of caracaras and the study site.

## 2 MATERIALS AND METHODS

### 2.1 Study area

Fieldwork took place during the austral spring (i.e., breeding season, November–December, 25–45 days/year) in 2014, 2016, and 2017 and during fall (i.e., nonbreeding season, May, 20 days) 2017 in Franklin Bay at the southwestern coast of Isla de los Estados (Staten Island), Argentina (54°85′30S, 64°83′90W). The island is 540 km^2^ and is separated from the Tierra del Fuego Main Island by the 24 km wide Le Maire Strait. The climate is cold, humid, and oceanic, with winds mainly from the SW and a variable rainfall regime, ranging from 700 to 2,900 mm/year depending on the site (Morello et al., 2012). Mixed forests of Evergreen beech (*Nothofagus betuloides*) and Winter's bark (*Drymis winteri*) cover most of the island, but along the fjords and coasts, a grassland vegetation typical of Subantarctic islands is present. There are over 100 species of birds and several marine mammals. The island is an important site for South American Sea lions (*Otaria flavescens*) and Fur seals (*Arctocephalus australis*), both of which are recovering from past exploitation (Milano et al., 2020a, 2020b). There are no native terrestrial mammals apart from the Chuanisín mouse (*Abrothrix* [*Angelomys*] *xanthorhina*), but rats (*Rattus sp*.), feral goats (*Capra hircus*), and red deer (*Cervus elaphus*) are commonly observed introduced species (Massoia & Chebez, 1993).

Within its tussock (*Poa flabellata*) grasslands, our 4 km^2^ study site at Franklin Bay holds a large southern rockhopper penguin colony with 127,000 breeding pairs, plus 4,600 breeding pairs of imperial shag (*Leucocarbo atriceps)* and 1,600 Magellanic penguin (*Spheniscus magellanicus)* breeding pairs (Raya Rey et al., 2014). Grazing pressure by introduced herbivores (goats since 1856 and red deer since 1974) has apparently restricted nest site availability for caracaras, and some rockhopper penguin subcolonies have no associated caracara nests (Balza et al., 2017). Caracaras are the most abundant scavenger on the island (Frere et al., 1999), being over six times more abundant than the second most abundant species (i.e., southern crested caracara, *Caracara plancus*, UB unpublished).

### 2.2 Pellet analyses

On Isla de los Estados, caracaras are the most important predator of rockhopper penguin chicks (Liljesthröm et al., 2008), but no information on other prey items was available. To establish the prey items potentially included in the stable isotope mixing model analysis (see below), we analyzed pellets from nests and identified their remains. This technique is biased over prey that leave hard remains (e.g., hairs, feathers, exoskeletons), and, as in our case, are generally encountered in the vicinity of nest sites (Marti et al., 2007; Redpath et al., 2001). Each year, we searched for active caracara nest sites by walking systematically through the study area and observing territorial behavior of breeding caracaras (for details see Balza et al., 2017). The number of accessible, active nests found in each year was 11–13 and represented ~70% of the observed breeding population. At first observation, the caracaras were in either the late incubation or early chick rearing stage. Pellets were dried and analyzed following Marti et al. (2007) and Rexer‐Huber and Bildstein (2013). Hairs in pellets were identified following Chehébar and Martín (1989) complemented with a reference collection for deer and goats from the study area. Feathers, eggs, and bones were identified with a reference collection of adults, chicks, and eggs from the breeding species listed above as well as geese (*Chloephaga picta*) and gull species that also breed on Isla de los Estados.

### 2.3 Blood and feather collection

Blood samples (~1 ml) were collected from the brachial vein of ~20‐day‐old chicks (43 individuals from 17 nest sites; 1–3 chick/nest*year) captured manually, and from juveniles, immatures, and adults during the breeding (*n* = 8) and nonbreeding (*n* = 8) seasons captured with walk‐in and noose traps, and later stored in 70% ethanol (Hobson et al., 1997). Age of individuals was determinate by plumage cues (Strange, 1996). We used the mean value of each nest for those with more than one chick, obtaining 8–10 independent samples/year. Also, as in some cases we collected samples from the same nests in multiple years, when we estimate overall isotopic niche parameters for chicks, we use the mean isotopic values for each one of the 17 nest sites. All 59 captured birds were banded with plastic rings (Ecotone, Poland), and no individual was sampled twice during the study period. To obtain floater and breeding adult samples, we collected molted wing feathers and classified them in relation to their distance from the nests. When collected from nest sites, we assumed it was molted by a breeding adult (*n* = 13, one feather/nest); and when collected >300 m apart from any active nest, by a floater (*n* = 63). Caracaras nest in a nearly colonial arrangement with very small breeding territories (Strange, 1996). The >300 m threshold was assumed not likely to represent breeding adult samples because observed foraging of the breeding adults was mainly associated with the nearest penguin patch (i.e., median < 50 m and in all cases < 200 m) and floaters are two‐ to fivefold more abundant than breeding adults (UB unpublished). Therefore, we assume a distance of >300 m from any known nest site is an area unlikely to be used by a breeding adult. Feathers were identified as belonging to adult birds (i.e., >5 years old) following Strange (1996). Floater abundance was 92 (95% CI 62–139) individuals in 2018, and since we obtained 11–29 samples/year, we assume no double sampling in this part of the population either. Molting of feathers in the study area was only observed during the breeding season for both floaters and breeding adults, and thus, we assumed that feathers are synthetized during the period of rockhopper penguin presence. Samples used are summarized in Table 1.

**TABLE 1:** Summary of caracara's samples used in this study to estimate isotopic niche metrics and stable isotope mixing models

Trophic category	Tissue sampled	Group	*n*	Aim
Selective	Blood	Juveniles, immatures, and adults in breeding season	8	Isotopic niche width estimation and mixing model analysis
Opportunistic		Juveniles, immatures, and adults in nonbreeding season	8	
Selective		Chicks (nests)	8−10/year	
Selective	Molted wing feathers	Breeding adults	13	
Opportunistic		Floaters	11−29/year	

### 2.4 Prey sample collection

To describe the potential prey resources for caracaras for building mixing models, we collected tissue samples of representative prey based on prey remains observed in pellets, published literature, and field observations (Catry et al., 2008; Rexer‐Huber & Bildstein, 2013). From 2017 to 2018, we collected samples from marine and terrestrial prey on Isla de los Estados (Table 2). Mussels were collected manually from the intertidal during low tide. Birds and invasive mammal samples were collected from fresh dead animals in our systematic surveys along the shores and at seabird colonies. Recently abandoned eggs were collected manually. Rodents were collected using Sherman‐like traps, and insects were collected using pitfall traps. Sea lion feces observed to be eaten by caracaras were collected in the nonbreeding season Observatorio Island, 40 km to the NE of our study area. All other samples were collected in Franklin Bay during the breeding season.

**TABLE 2:** Prey samples and their δ^15^N and δ^13^C values (‰, mean ± *SD*) used to build stable isotope mixing models for caracaras in Franklin Bay, Isla de los Estados, Argentina. Asterisk denotes non‐native species

Trophic web	Species	Tissue	*n*	δ^15^N	δ^13^C
Marine	Mussel (*Mytilus edulis*)	Muscle	5	10.8 ± 0.6	−14.7 ± 0.3
	Rockhopper penguin (egg)	Egg membrane	3	9.6 ± 0.4	−21.4 ± 0.5
	Rockhopper penguin (chick)	Muscle	11	9.7 ± 2.5	−21.8 ± 1.7
	Rockhopper penguin (adult)	Muscle	2	8.0	−22.9
	Imperial shag (egg)	Egg membrane	3	15.7 ± 0.7	−15.1 ± 0.4
	Imperial shag (chick)	Muscle	6	14.8 ± 1.0	−16.8 ± 0.4
	Sea lion	Feces	3	15.4 ± 0.8	−18.8 ± 0.8
Terrestrial	Red deer*	Muscle	3	4.6 ± 4.4	−24.9 ± 0.1
	Goat*	Muscle	1	3.3	−24.7
	Goose (*Chloephaga picta)*	Muscle	2	12.2	−30.4
	Rat (*Rattus rattus*)*	Muscle	3	9.5 ± 4.7	−19.8 ± 4.2
	Chuanisín mouse	Muscle	3	4.7 ± 2.3	−23.9 ± 2.2
	Beetle (*Ceroglossus suturalis*)	Muscle	6	5.7 ± 5.2	−27.9 ± 0.8

### 2.5 Stable isotope analysis

Stable isotope (SI) analysis provides useful insights on species trophic ecology that avoids many of the biases of traditional diet study methods (Bearhop et al., 2004). Isotopic ratios for those elements that are incorporated through diet can be interpreted as a reflection of consumer's food webs pathways (Chisholm & Nelson, 1982; Hobson & Clark, 1992a) and trophic level (Minagawa & Wada, 1984). However, consumer SI values also reflect spatial and temporal variation in food sources' SI values and thus are not necessarily equivalent to niche variation (Matthews & Mazumder, 2004). Also, the SI values of consumer tissues are context dependent, and quantifying baseline information is important when applying this technique in new study sites/species (Phillips et al., 2014).

SI analysis allows testing hypothesis of OFT for two reasons: First, it provides quantitative, individual‐level and temporally integrated data. Therefore, diet variation considers temporal consistency and is not a snapshot of the diet of individuals (Novak & Tinker, 2015). Second, intraspecific variation in resource use is reflected by shifts in consumer tissue isotope ratios in a predictable way (Hammerschlag‐Peyer et al., 2011). Additionally, SI mixing models can be useful to detect the importance of prey such as carrion that are not well represented in classic techniques.

To prepare our samples, we rinsed feathers with a 2:1 chloroform:methanol solution to remove surface lipids and dried them at room temperature. Blood samples were first dried at 60°C for 24 hr and then freeze‐dried for another 24 hr. We weighed ~0.60 mg of each sample into tin capsules, which were flash combusted in a Costech ECS4010 elemental analyzer coupled to a Thermo‐Fisher Delta Plus XP continuous‐flow stable isotope ratio mass spectrometer. Stable isotope values were normalized using a two‐point system with glutamic acid reference material (USGS‐40 and USGS‐41). Measurement precision based on reference material was 0.1‰ for both δ^13^C and δ^15^N. Stable isotope values were calculated with the following equation and are expressed in standard delta (δ) notation in per mil units (‰):

δX = [(*R*_sample_/*R*_standard_) − 1]×1,000

where X is ^13^C or ^15^N and *R* is the corresponding ratio ^13^C/^12^C or ^15^N/^14^N. The *R*_standard_ values were based on Vienna Pee Dee Belemnite (VPDB) for δ^13^C and atmospheric N_2_ (AIR) for δ^15^N values.

Since physiological, tissue‐dependent traits are known to be relevant for isotopic ratios (Hobson & Clark, 1992b), we needed to assume the differential factors acting for different tissues. As a consistent linear relation between blood and feather samples in bird chicks with marine diets exists, we normalized blood stable isotope values to reflect feather stable isotope values (Cherel et al., 2014), to further compare blood from chicks with feathers of breeding adults and floaters.

### 2.6 Isotopic niche metrics and statistics

We compared isotopic niche width and overlap among groups to test hypotheses regarding trophic expansion/reduction dynamics and changes in resource use between seasons (Hammerschlag‐Peyer et al., 2011). We first quantified isotopic niche width, which is a common metric used to quantify variability in trophic diversity and resource use (Bearhop et al., 2004; Newsome et al., 2007). We calculated Bayesian standard ellipse areas (SEA_B_) using the SIBER package in R software (Jackson et al., 2011; R Core Team, 2018). SEA_B_ are iteration‐produced, posterior probabilities of the 2‐dimensional isospace of the groups that allow comparison between unbalanced sample sizes (Jackson et al., 2011). For each model, we ran 10,000 Markov chain Monte Carlo iterations, discarding the first 1,000 of the analysis with default priors. For posterior comparisons, we tested the probability of one group's SEA_B_ being bigger than the other group by comparing the proportion of posterior ellipses (PP) that differed between groups. We considered PP ≥ 0.95 to reflect relevant differences in SEA_B_. Interannual variation was studied for chicks and floaters only, because the breeding adult sample size was too low, and thus, only a pooled analysis was used for them.

To compare overlap in resource use among groups and between seasons, we estimated the probability of individuals in one group to be contained in the ellipses of another using the nicheROVER package (Swanson et al., 2015). Overlap values range from 0 (i.e., no overlap) to 1 (i.e., complete overlap). To test the occurrence of nested patterns, we looked for differences in the 95% credible intervals (CI) of the estimations among reciprocals. For example, for supporting the hypothesis of group A being a subset of group B, we looked for asymmetry in overlap, meaning that individuals in group A are more likely to be encompassed in the ellipses of group B than vice versa.

### 2.7 Mixing model analysis

We built Bayesian stable isotope mixing models using the MixSIAR package (Stock et al., 2018). We separated our caracara samples into groups according to age, season, and breeding status. Stable isotope mixing models can be sensitive to the trophic discrimination factors (TDF) used (Bond & Diamond, 2011), and having the consumer data included in convex hulls is a necessary though insufficient condition for mixing models to work properly (Phillips et al., 2014). For this, we used the method described by Smith et al. (2013) to simulate stable isotope mixing polygons and to select TDF sources that would allow for a suitable mixing model. Depending on the tissue and age class considered, we contrasted up to four TDF sources: from a related species (peregrine falcon, Hobson & Clark, 1992b), from a scavenger bird of prey (California condor, Kurle et al., 2013), from a subpolar raptor (snowy owl, Therrien et al., 2011), and from a meta‐analysis‐derived TDF using the SIDER package (Healy et al., 2017) (Table S1). We ran the mixing models with all suitable TDF to explore possible effects on the election of TDF on final output (Figure S2, Table S4).

Also critical to the performance of mixing models is the election of priors. Informative priors are recommended, when information is available, to constrain the output of indeterminate models (Phillips et al., 2014). They can accurately describe the diet input in some cases (Chiaradia et al., 2014), but they can also produce poor model performance when pellet/scat analysis are used, because they tend to overestimate the importance of prey with indigestible parts (Swan et al., 2020). In our case, we first used pellet analysis to constrain the selection of potential prey for breeding adults and their chicks, assuming that potentially important prey types should occur at least once in this analysis (Table 3). Then, we used informative priors based on abundance of prey types for breeding season models only, which were available in published works for seabirds (Raya Rey et al., 2014) and our own estimations for geese (UB unpublished). Rockhopper penguins are 27‐ and 85‐fold more abundant than shags and geese, respectively, in our study area. We set our informative priors to reflect a 5% minimum importance of all prey types other than rockhopper penguins, therefore setting a precautionary underweighting of rockhopper's signature in the starting point of the models. For the nonbreeding season model, we used uninformative priors because of the lack of more detailed information. Deer and goat were combined in one signature as we had only one sample of the latter. The potential prey used in each model are detailed in Table 3. Following Phillips et al. (2014), we combined sources a posteriori into “terrestrial” and “marine” to distinguish between these two trophic pathways. Because marine and terrestrial prey were not evenly sampled (e.g., for the nonbreeding, season four terrestrial sources and two marine sources were used), even the “uninformative” prior models were informative of marine and terrestrial input (see Phillips et al., 2014). Depending on the model, initial terrestrial input varied from 9% to 67% (Table S4). For chick's models, we used nest id as random effects. We ran all our models with 300,000 Monte Carlo iterations. We checked whether the models converged with two different diagnosis statistics (Stock et al., 2018), and we informed all plausible models and ranked them using deviance information criteria (DIC, Ando, 2010) (Table S4).

**TABLE 3:** Marine and terrestrial prey signatures used in stable isotopes mixing models

Trophic category	Model (group, tissue)	Marine signature components	Terrestrial signature components	Justification
Selective	Chicks, Blood; Breeding adults, wing feathers	Rockhopper penguin and imperial shag (eggs, chicks and adults)	Insects and geese	No evidence of terrestrial prey other than insects and geese by pellet analysis (Table 3). Although caracaras can also predate chicks of the other breeding seabird in the area, the Magellanic penguin (K. Harrington com. pers.), we have no records of such behavior in our site (see Results, pellet analysis) and this species is the less abundant seabird breeding in the study area. Therefore, we assume its importance to be no significant
Opportunistic	Floaters, wing feathers		Insects, geese, deer, goat, and rodents	Uncertainty about prey taken; all observed and potentially important prey sources included in terrestrial items
Selective	Breeding season, blood			
Opportunistic	Nonbreeding season, blood	Sea lion feces and mussels	Deer, goat, insects, and rodents	No seabirds and geese available during nonbreeding season. Association with pinniped feces are “probably the most important source of food in the feeding cycle of *Phalcoboenus* in the winter” (Strange, 1996), and caracaras feeding on sea lion excreta were observed in nearby Observatorio Island in the nonbreeding season, where the sea lion samples were collected. Bivalves observed as prey in other populations (Catry et al., 2008; Rexer‐Huber & Bildstein, 2013) and in our case, although available all‐year round, are considered potentially important only in the absence of seabird colonies

## 3 RESULTS

### 3.1 Pellet analysis

We analyzed 138 pellets from 19 nest sites, (mean 4.6 pellets/site‐year, range: 1–15). Penguins, insects, and eggshells were the most frequent prey items found, and no mammal remains were encountered (Table 4). Ten adult penguin feathers were identified at the species level, and they all belonged to rockhopper penguins. 97% of eggshells corresponded to seabirds, and the other 3% were identified as corresponding to Upland goose.

**TABLE 4:** Frequency of occurrence of prey items in caracara pellets collected at 19 nest sites (in parenthesis for each year) in Franklin Bay, Isla de los Estados

Class	Common name	Scientific name	Frequency of occurrence (%)			
			2014 (8)	2016 (9)	2017 (13)	Overall (19)
Birds	Penguin (adult)	Spheniscidae	60.4	84.8	76.9	73.2
	Penguin (chick)		37.7	8.7	28.2	25.4
	Upland goose (adult)	*Chloephaga picta*	1.9	0	5.1	2.2
	Imperial shag (adult)	*Leucocarbo atriceps*	0	0	2.6	0.7
	Eggshell	Aves	22.6	21.7	38.5	26.8
Mammals	Feral goat	*Capra hircus*	0	0	0	0
	Red deer	*Cervus elaphus*	0	0	0	0
	Rat	*Rattus sp*.	0	0	0	0
	Chuanisín mouse	*Abrothrix (Angelomys) xanthorhina*	0	0	0	0
Insects	Beetles	Coleoptera (mostly *Ceroglossus suturalis*)	71.7	41.3	59.0	58.0
Plants	Tussock grass	*Poa sp*.	81.1	69.6	76.9	76.1
	Rush	Juncaceae	26.4	34.8	38.5	32.6
	Seeds		0	0	5.1	1.4
Sponges		Porifera	0	2.2	2.6	1.4
					
Inorganic	Pebbles (5–15 mm)		18.9	37.0	41.0	31.2
	Plastic		0	0	2.6	0.7

### 3.2 Isotopic niche analysis

**FIGURE 2:**
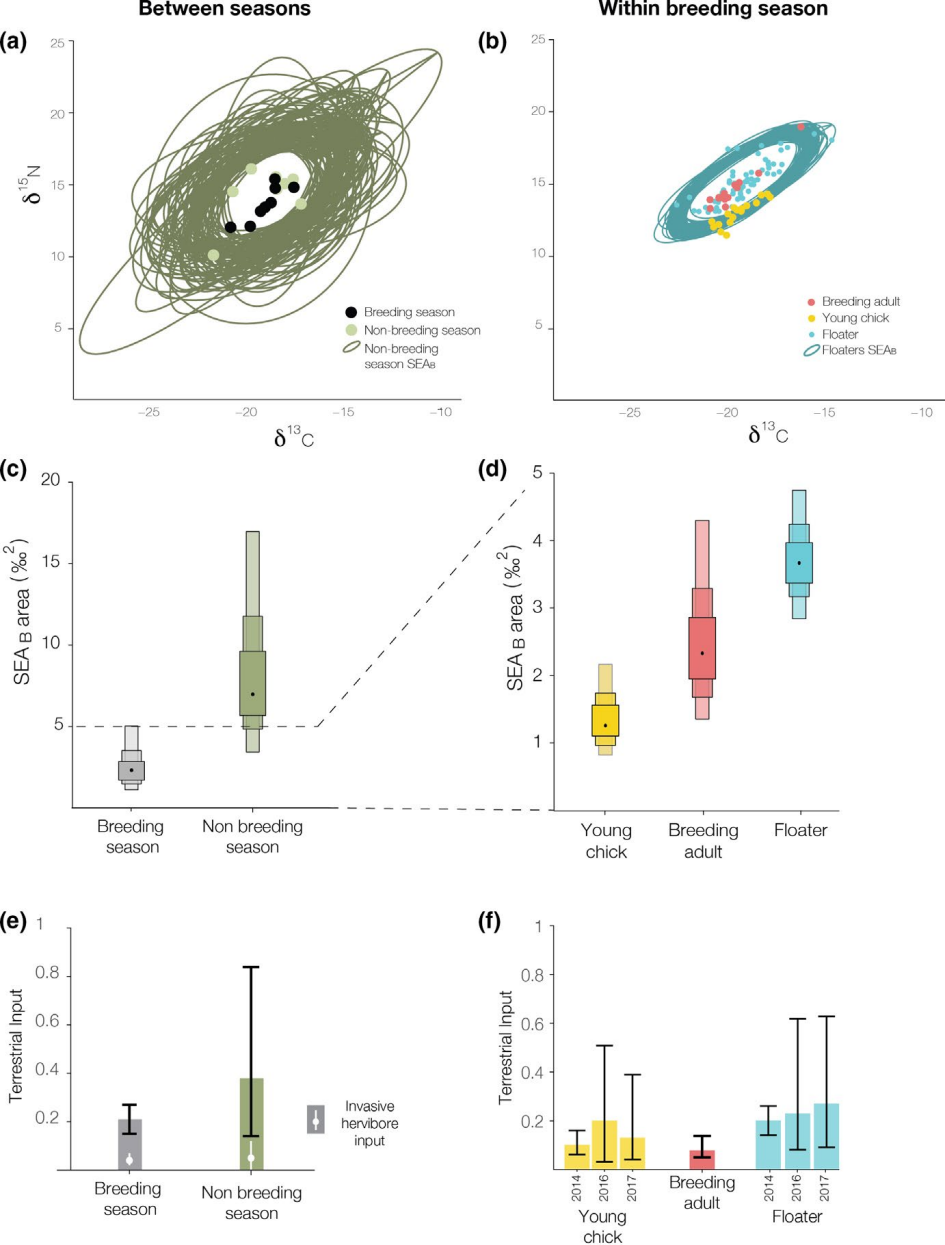
Intraspecific diet variation in striated caracaras in Isla de los Estados estimated through stable isotope analyses. In each row, the seasonal model is shown on the left and the within‐breeding season model on the right. (a, b) Nested pattern of intrapopulation stable isotope ratios of striated caracaras. Bayesian standard ellipses are shown for the opportunistic groups in each case, and dots represent selective groups' raw signatures. (c, d) Size of Bayesian standard ellipse areas. Black dots represent mode, while boxes represent 50%, 75%, and 95% credible intervals. (e, f) Stable isotope mixing model posterior distribution of terrestrial input. Bars represent median and error segment the 95% credible interval. Best model for each group is shown, but output virtually did not differ between different suitable TDFs (see Table S4). We also show invasive herbivores contribution in breeding and nonbreeding season in white (median and 95% credible interval, (e) within the overall terrestrial estimate

A nested pattern in the isotopic niche was observed both between seasons and within the breeding season. Overlap estimates showed that blood from the breeding season was more likely to be enclosed within blood from the nonbreeding seasons' ellipses than vice versa (Figure 2a, Table S3). Within the breeding season, breeding adults and chicks were more likely to be enclosed within the floaters' ellipses than vice versa and showed virtually no overlap between their ellipses (Figure 2b, Table S3).

Isotopic niche width was threefold larger during the nonbreeding season than during the breeding season (PP = 0.99, *n* = 8 for each season). (Figure 2c, Table S2). During the breeding season, isotopic niche width of floaters was almost threefold larger than chicks' (PP ~ 1, *n* = 17–63), but it did not differ from breeding adults (PP = 0.88, *n* = 13–17, Figure 2d). Breeding adults isotopic niche width was larger than chicks' (PP = 0.95, *n* = 13–63). (Figure 2d, Table S2).

Interannual comparisons (only possible for the breeding season) showed overlap between years for both floaters and chicks (Table S3). Chicks' isotopic niche width did not differ between years, but for floaters, however, 2016 had approximately two times the isotopic niche width of 2014 and 2017 (Table S2).

### 3.3 Mixing model analysis

Two or three TDFs were suitable for describing our data, depending on the group analyzed (Figure S1). However, despite showing differences in model fit according to DIC, the effect of different TDFs in prey proportion was not important, showing all of them to have virtually equal outputs (Table S4). Overall, we observed marine prey to be consistently the most important food input source across all models (62%–91%, Figure 2e,f). During the nonbreeding season, when the total estimated terrestrial supply was 38%, invasive mammals accounted for only 5% (95% CI: 0%–23%) of the input (Figure 2e).

## 4 DISCUSSION

Our findings suggest that caracaras at Franklin Bay possess a dynamic trophic niche associated with mobility restrictions and seasonal pulses in the abundance of rockhopper penguins. We found higher variation in trophic niche during the nonbreeding season for the population as a whole and for floaters within the breeding season, two groups a priori classified as opportunistic. At the population level, it appears caracaras at Franklin Bay are not “true” specialists, but instead facultative specialists whose level of specialization can vary with seabird availability and individual breeding status.

Stable isotope mixing model analysis suggests that broader isotope niches, characteristic of opportunistic groups, are not necessarily related to the incorporation of terrestrial prey (Figure 2e,f). Spatially flexible individuals could diversify their trophic choices mainly within marine resources as previously described for the Malvinas/Falklands population (Rexer‐Huber & Bildstein, 2013; Strange, 1996). Even during the nonbreeding season, when the estimated terrestrial supply was around 40%, deer and goats accounted for only 5% of the input. These results, together with pellet analysis and our personal observations, provide no evidence to support a relevant subsidy catered by invasive herbivores for caracaras at this site.

Trophic variation following changes in prey availability has been documented in other raptor species (e.g., Moleón et al., 2012; Nadjafzadeh et al., 2016). According to optimal foraging theory, extended by the Shared Preferences model, the niche of the most selective individuals becomes a subset of the niche of opportunistic ones (Svanbäck & Bolnick, 2005). Assuming that the seasonal pulse of the nearly 300,000 penguins and their products (i.e., eggs and chicks) are the top‐ranked prey in our study area, then the breeding season niche should be smaller and included within the nonbreeding season's niche. Within the breeding season, territorial behavior provides breeding adults and their chicks higher, but still nonexclusive, access to penguin subcolonies. This fact, together with spatial restrictions associated with chick rearing duties would drive their smaller isotopic niche to be nested within floaters' niche.

Our results are in general consistent with the Shared Preferences model, but we can outline two other, nonexclusive explanations for the observed pattern. First, individuals could have different optimal diets according to season, age, and breeding status. In our case, juveniles/immatures are more represented in the nonbreeding season sampling (five out of eight blood samples) than in the breeding season (three out of eight). Thus, if caracaras acquire foraging skills with age, it is possible that the observed population level niche expansion during the nonbreeding season could reflect juvenile inexperience (Wunderle, 1991). In the description of the models (Svanbäck & Bolnick, 2005), individual phenotypes (i.e., different handling abilities) are the drivers for different optimal diets. In our case, floaters could maximize their fitness by reducing competition with conspecifics, if survival threshold can be reached by feeding on alternative prey. Our results also show differences in isotopic niche dimensions between breeding adults and chicks. In other raptors, small niche width in chicks relative to their parents has been observed (Catry et al., 2016), and breeding success can be positively related to low diet variation in chicks (Otterbeck et al., 2015). In our study system, breeding adults could have foraged opportunistically in general, but behaved as specialists when feeding their offspring. For instance, breeding adults might choose to feed on certain items that are available even within their restricted foraging area, but which are not delivered to chicks, such as eggs or carrion (Newton, 1979). This strategy could avoid preferred prey depletion around the nest area and also explain the low isotopic niche overlap between breeding adults and chicks, as observed at least in one other raptor species (e.g., Catry et al., 2016).

Another possibility is that individuals may simply not behave optimally. Pierce and Ollason (1987) argued, among other criticisms to OFT, that optimality does not necessarily occur in nature, mainly because of two reasons. On the one hand, genetic restrictions limit the variation available for natural selection to operate on. On the other hand, ecological change constantly redefines what an optimal individual is (Pierce & Ollason, 1987). In our study system, interference competition could impede some individuals from reaching their optimal diet. Larger niche width of floaters can be attributed to three, nonexclusive factors: (a) an expansion of their trophic niche to include nonpenguin resources, (b) their greater foraging mobility, and (c) opportunistic and/or differential accessibility to the penguin colony due to agonistic/hierarchical interactions between individuals. The latter has been found in Malvinas/Falklands other populations (Autilio et al., 2019), in which adults had higher hierarchical ranks relative to immatures and juveniles in competing for carcasses. Other caracara species are also known for their complex social behaviors that restrict or enhance the foraging niche of an otherwise isolated individual (e.g., Biondi et al., 2010; Jones, 1999; Thiollay, 1991).

### 4.1 Limitations

Information on caracara diet in other populations is available (Catry et al., 2008; Rexer‐Huber & Bildstein, 2013), and some of their potential prey items' abundance have been assessed in our study area (Raya Rey et al., 2014). However, when accounting for stable isotopes mixing models, some simplifications of the system were needed. Trophic discrimination factors have been thoroughly discussed as potential sources of error in SI studies (Bond & Diamond, 2011). Also, our models assumed no spatiotemporal variation in prey signatures and no variation in tissue‐specific turnover rates since contemporary sampling of both consumers and preys was not possible due to logistical reasons. In our study site, temporal variation in isotopic ratio in seabirds was observed (Harris et al., 2016; Rosciano et al., 2018, 2019). However, interannual differences observed (especially in δ^13^C) were lower than the differences we observed for marine and terrestrial prey items. We also found that the SI values of rats were within the range of values found in marine sources in our system. The subsidy of marine nutrients to terrestrial food webs is a well‐known phenomenon (e.g., Bastow et al., 2002; Bouchard & Bjorndal, 2000; Bump et al., 2009; Catenazzi & Donnelly, 2007) and can be observed from the isotopic ratio viewpoint (e.g., Bokhorst et al., 2019). Rats on other Subantarctic islands show strong spatiotemporal variation in diet choices, feeding regularly on marine prey (Quillfeldt et al., 2008), which could add additional challenges when interpreting our results.

Finally, during the nonbreeding season, there is little record of caracara movement and foraging behavior, which may hide other factors driving intraspecific variation during this time of the year. Field observations indicated around one order of magnitude of fewer individuals during the nonbreeding season in Franklin Bay, and records of banded and GPS‐attached individuals show that, although apparently restricted to the island year‐round, they can move around its entire area within a few days (UB unpublished).

### 4.2 Concluding remarks

Specialization of striated caracara over the seasonal pulse of seabirds could have arisen at the regional level driven by the geographical restriction posed by the last glaciations (Meiburg, 2006; Vuilleumier, 1991). Here, we present evidence of individual diet variation driven by ecological context (Novak & Tinker, 2015). We do not provide evidence of specialization in adaptative terms, but only apparent, facultative specialization associated with preferred prey availability (Devictor et al., 2010; Pagani‐Núñez et al., 2016). We found that two processes seem to be involved in the availability of the preferred resource: on the one hand, the pulsed event of penguin resources and the territorial behavior of the breeding adults; and on the other hand, mobility restrictions related to chick rearing during the breeding season. The relationship between mobility and resource use is rare in the literature (but see Urton & Hobson, 2005; Webber et al., 2020). In the case of caracaras on Isla de los Estados, the spatial restriction posed by the seasonal pulse of rockhopper penguins and the reproductive status of individuals correspond to stable isotope values. The niche expansion observed in groups with less access to seabird subcolonies may not reflect increased foraging on terrestrial prey, but rather be driven by a greater variation in the types of marine prey items consumed.

## CONFLICT OF INTEREST

The authors declare no conflict of interest.

## AUTHOR CONTRIBUTIONS

**Ulises Balza:** Conceptualization (equal); data curation (lead); formal analysis (lead); investigation (equal); methodology (equal); writing – original draft (lead); writing – review and editing (equal). **Nicolás A. Lois:** Conceptualization (equal); data curation (equal); investigation (equal); visualization (lead); writing – review and editing (equal). **Michael J. Polito:** Data curation (supporting); formal analysis (supporting); methodology (supporting); supervision (supporting); writing – review and editing (equal). **Klemens Pütz:** Funding acquisition (equal); investigation (equal); supervision (supporting); writing – review and editing (equal). **Amira Salom:** Conceptualization (supporting); data curation (equal); visualization (supporting); writing – review and editing (equal). **Andrea Raya Rey:** Conceptualization (equal); funding acquisition (equal); investigation (equal); methodology (equal); project administration (lead); supervision (equal); writing – review and editing (equal).

### Open Research Badges

This article has earned an Open Data Badge for making publicly available the digitally‐shareable data necessary to reproduce the reported results. The data is available at https://doi.org/10.5061/dryad.n2z34tmv4.

## Supporting information

Appendix S1

## Data Availability

Stable isotopes raw data analyzed in this work are available in Dryad under the https://doi.org/10.5061/dryad.n2z34tmv4. Corrected δ15N and δ13C and C/N ratio is reported.
