# Supplementary material for: The dynamic trophic niche of an island bird of prey
Source: Ecol Evol. 2020 Oct 3;10(21):12264–76. doi: 10.1002/ece3.6856 (PMC7663050; doi:10.1002/ece3.6856)
Supplement: Supplementary file 1 — Appendix S1 [file ECE3-10-12264-s001.docx]

**Appendix S1**

**Supplementary figures and tables**


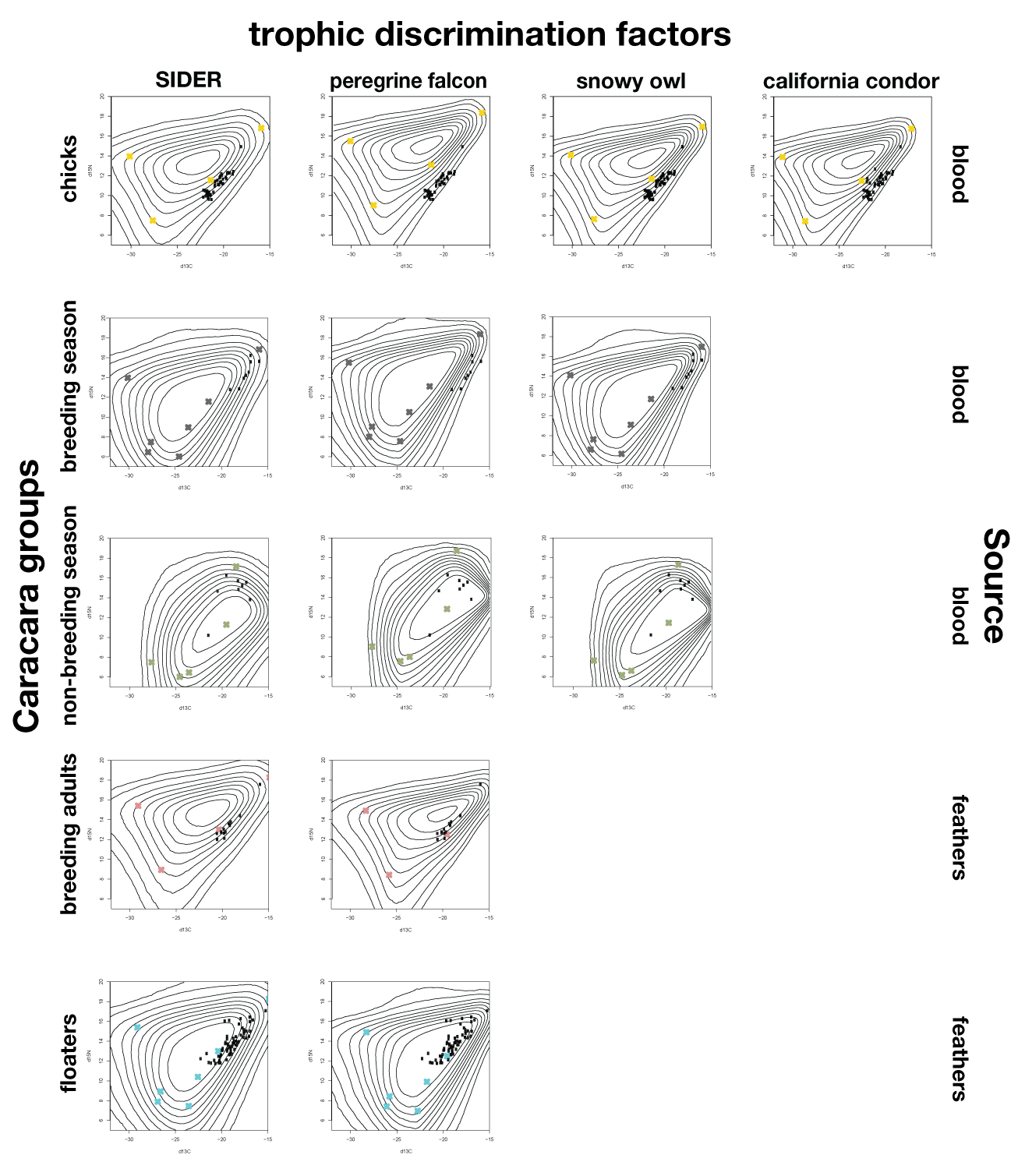


**Figure S1.** Stable isotope mixing model polygons for striated caracara tissues relative to potential prey, following Smith et al. (2013). In the top lies the TDF used, in the left lies the caracara group, and in the right lies the tissue analysed. Black dots represent consumer signature and color-coded crosses (following the criteria of Figure 1) represents average source signatures corrected for corresponding TDF. Lined-region represents 95% confidence intervals, each line representing 5%. When some consumers lied outside the lined region, the mixing model with that TDF was not conducted (Table S4).


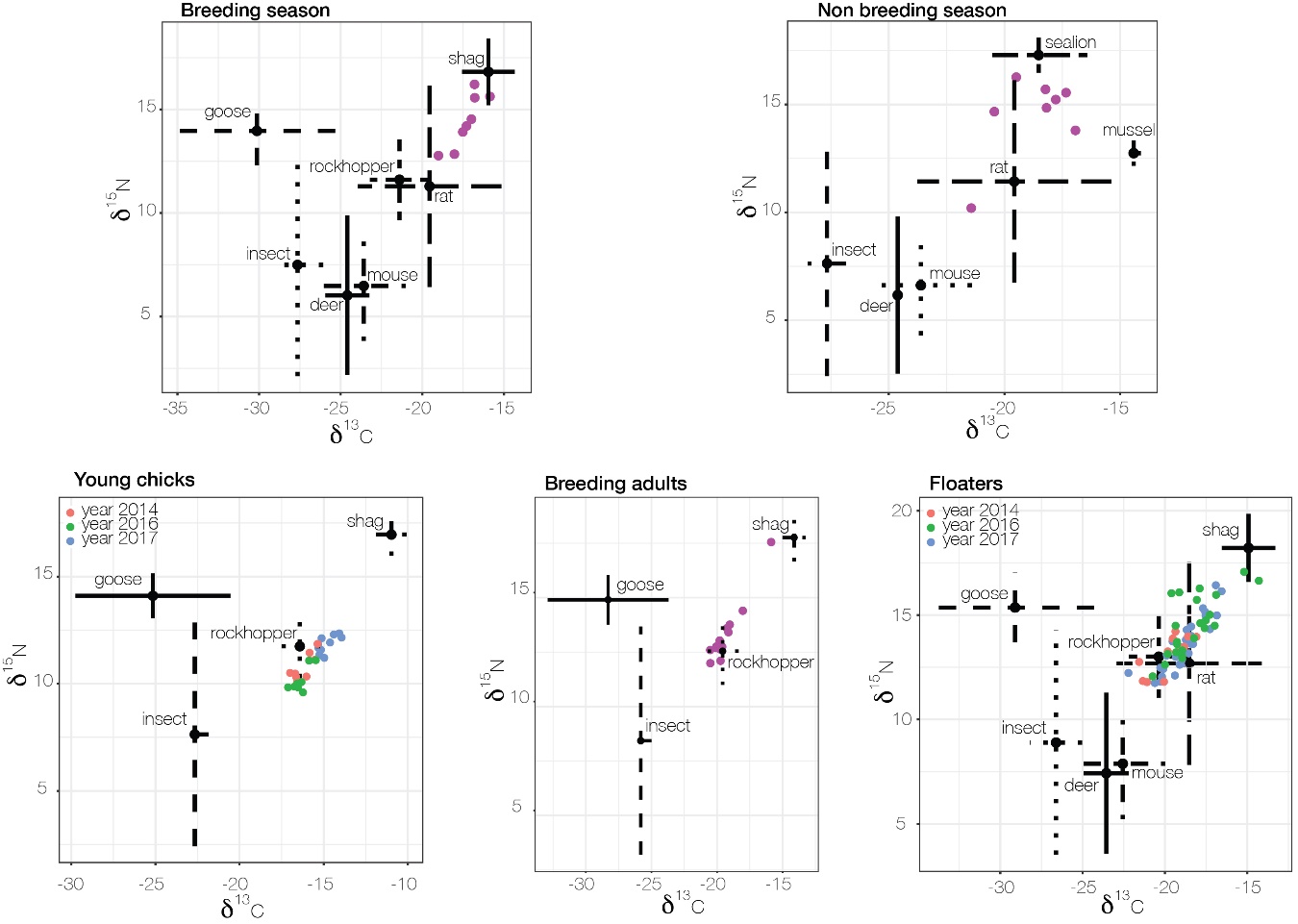


**Figure S2.** An example of input for mixing model analysis, showing the different marine and terrestrial sources’ signatures (lines) for each group of consumers (points), constructed with data of Table 2 and according to decisions justified in Table 3. TDFs used for this example are snowy owl’s for blood samples (chicks and non-breeding season) and SIDER-derived for feather samples (breeding adults and floaters).

**Table S1.** Trophic discrimination factors (TDF) tested in striated caracara mixing models.

| TDF | Tissue | ΔN (‰ ± SD) | ΔC (‰ ± SD) | Source |
| --- | --- | --- | --- | --- |
| Peregrine falcon | Whole blood | 3.3 ± 0.4 | 0.2 ± 0.01 | Hobson *et al.* (1992)b |
|  | Feathers | 2.7 ± 0.5 | 2.1 ± 0.08 |  |
| Snowy Owl | Whole blood | 1.9 ± 0.2 | 0.3 ± 0.1 | Therrien *et al*. (2011) |
| California condor | Whole blood (chicks) | 1.7 ± 0.1 | -0.7 ± 0.1 | Kurle *et al.* (2013) |
| SIDER package | Feathers | 3.2 ± 1.3 | 1.3 ± 1.4 | Healy *et al.* (2017) |
|  | Whole blood | 1.8 ± 1.3 | 0.3 ± 1.3 |  |

**Seasonal model**

|  | A |  |
| --- | --- | --- |
| B | **Breeding season** | **Non-breeding season** |
| Breeding season | **-** | 0.99 |
| Non-breeding season |  | **-** |

**Ontogenic model**

|  | **A** | | | |
| --- | --- | --- | --- | --- |
| **B** |  | **Chick** | **Breeding adult** | **Floater** |
|  | **Chick** | - | 0.95 | 0.9998 |
|  | **Breeding adult** |  | - | 0.88 |
|  | **Floater** |  |  | - |

**Inter-annual models for chicks (a) and floaters (b)**

| **(a)** | **A** | | | |
| --- | --- | --- | --- | --- |
| **B** |  | **2014** | **2016** | **2017** |
|  | **2014** | - | 0.40 | 0.78 |
|  | **2016** |  | - | 0.22 |
|  | **2017** |  |  | - |
| **(b)** | **A** | | | |
| **B** |  | **2014** | **2016** | **2017** |
|  | **2014** | - | 0.97 | 0.63 |
|  | **2016** |  | - | 0.02 |
|  | **2017** |  |  | - |

**Table S2.** Probability of isotopic niche area in group A is bigger than the group B for each model.

**Seasonal model**

|  | A |  |
| --- | --- | --- |
| B | **Breeding season** | **Non-breeding season** |
| Breeding season | **-** | 0.39 (0.19-0.66) |
| Non-breeding season | 0.97 (0.84-1.00) | **-** |

**Ontogenic model**

|  | **A** | | | |
| --- | --- | --- | --- | --- |
| **B** |  | **Chick** | **Breeding adult** | **Floater** |
|  | **Chick** | - | 0.01 (0.00-0.04) | 0.06 (0.02-0.12) |
|  | **Breeding adult** | 0.01 (0.00-0.06) | - | 0.50 (0.31-0.72) |
|  | **Floater** | 0.37 (0.13-0.69) | 0.96 (0.79-1.00) | - |

**Inter-annual models for chicks (a) and floaters (b)**

| **(a)** | **A** | | | |
| --- | --- | --- | --- | --- |
| **B** |  | **2014** | **2016** | **2017** |
|  | **2014** | - | 0.72 (0.31-0.97) | 0.37 (0.03-0.87) |
|  | **2016** | 0.71 (0.44-1.00) | - | 0.14 (0.00-0.84) |
|  | **2017** | 0.29 (0.02-0.71) | 0.09 (0.00-0.35) | - |
| **(b)** | **A** | | | |
| **B** |  | **2014** | **2016** | **2017** |
|  | **2014** | - | 0.53 (0.22-0.88) | 0.73 (0.42-0.96) |
|  | **2016** | 0.88 (0.65-0.99) | - | 0.93 (0.81-0.99) |
|  | **2017** | 0.80 (0.48-0.97) | 0.72 (0.49-0.86) | - |

**Table S3.** Mean and 95 % CI overlap between groups estimated as the probability of one individual in the group A falling into the isotopic niche of the group B.

| Model | TDF source | Tissue | Priors | Year | Initial terrestrial input (%) | Final terrestrial input (%, median, 95% CI) | DIC | ΔDIC |
| --- | --- | --- | --- | --- | --- | --- | --- | --- |
| Breeding season | SIDER | Blood | Informative |  | 21 | 0.21 (0.15-0.27) | 147.95 | 0 |
|  | Snowy owl |  |  |  |  | 0.20 (0.15-0.27) | 148.07 | 0.12 |
| Non breeding season | Snowy owl |  | Uninformative |  | 67 | 0.38 (0.14-0.84) | 100.43 | 0 |
|  | SIDER |  |  |  |  | Not converged | | |
|  | Peregrine falcon |  |  |  |  |  |  |  |
| Breeding adults | Peregrine falcon | Wing feathers | Informative |  | 9 | 0.08 (0.05-0.14) | 86.62 | 0 |
|  | SIDER |  |  |  |  | 0.08 (0.05-0.13) | 91.05 | 4.43 |
| Chicks | Snowy owl | Blood |  | 2014 |  | 0.10 (0.06-0.16) | 107.22 | 0 |
|  |  |  |  | 2016 |  | 0.20 (0.03-0.51) |  |  |
|  |  |  |  | 2017 |  | 0.13 (0.04-0.39) |  |  |
|  | SIDER |  |  | 2014 |  | Not converged | | |
|  |  |  |  | 2016 |  |  |  |  |
|  |  |  |  | 2017 |  |  |  |  |
| Floaters | SIDER | Wing feathers |  | 2014 | 21 | 0.20 (0.14-0.26) | 220.13 | 0 |
|  |  |  |  | 2016 |  | 0.23 (0.08-0.62) |  |  |
|  |  |  |  | 2017 |  | 0.27 (0.09-0.63) |  |  |
|  | Peregrine falcon |  |  | 2014 |  | Not converged | | |
|  |  |  |  | 2016 |  |  |  |  |
|  |  |  |  | 2017 |  |  |  |  |

**Table S4.** Mixing model outputs using all suitable trophic discrimination factors (TDF) according with method described in Smith *et al.* (2013) (see Figure S1). We compared all models using Deviance Information Criterion (DIC) and difference of DIC relative to the best model (ΔDIC). Only converged models showed DIC values. Median (95% CI) of terrestrial and marine proportion of diet are presented.

**Table S5.** Stable isotopes raw information analysed in this work. Corrected δ^15^N and δ^13^C and C/N ratio is reported.

| Species | Site | Year | Season | Tissue | Class | δ13C | δ15N | C/N Ratio |
| --- | --- | --- | --- | --- | --- | --- | --- | --- |
| Striated Caracara | Franklin | 2017 | Summer | Blood | Breeding adult | -19.54 | 12.22 | 3.36 |
| Striated Caracara | Franklin | 2017 | Summer | Blood | Breeding adult | -20.55 | 12.15 | 3.28 |
| Striated Caracara | Franklin | 2017 | Summer | Blood | Adult | -18.28 | 15.55 | 3.28 |
| Striated Caracara | Franklin | 2017 | Summer | Blood | Adult | -18.78 | 13.56 | 3.29 |
| Striated Caracara | Franklin | 2017 | Summer | Blood | Adult | -18.26 | 14.91 | 3.22 |
| Striated Caracara | Franklin | 2016 | Summer | Blood | Juvenile | -17.30 | 14.97 | 3.24 |
| Striated Caracara | Franklin | 2014 | Summer | Blood | Juvenile | -18.47 | 13.89 | 3.25 |
| Striated Caracara | Franklin | 2014 | Summer | Blood | Immature | -19.01 | 13.28 | 3.30 |
| Striated Caracara | Franklin | 2017 | Winter | Blood | Adult | -20.44 | 14.67 | 3.29 |
| Striated Caracara | Franklin | 2017 | Winter | Blood | Juvenile | -17.34 | 15.55 | 3.26 |
| Striated Caracara | Franklin | 2017 | Winter | Blood | Juvenile | -17.78 | 15.23 | 3.28 |
| Striated Caracara | Franklin | 2017 | Winter | Blood | Juvenile | -18.18 | 14.84 | 3.25 |
| Striated Caracara | Franklin | 2017 | Winter | Blood | Juvenile | -21.43 | 10.20 | 3.29 |
| Striated Caracara | Franklin | 2017 | Winter | Blood | Juvenile | -18.23 | 15.70 | 3.40 |
| Striated Caracara | Franklin | 2017 | Winter | Blood | Adult | -19.49 | 16.26 | 3.28 |
| Striated Caracara | Franklin | 2017 | Winter | Blood | Adult | -16.93 | 13.80 | 3.32 |
| Striated Caracara | Franklin | 2014 | Summer | Feathers | Floater | -18.83 | 13.48 | 3.42 |
| Striated Caracara | Franklin | 2014 | Summer | Feathers | Floater | -19.36 | 14.21 | 3.30 |
| Striated Caracara | Franklin | 2014 | Summer | Feathers | Floater | -18.60 | 13.98 | 3.27 |
| Striated Caracara | Franklin | 2014 | Summer | Feathers | Floater | -21.57 | 12.76 | 3.45 |
| Striated Caracara | Franklin | 2014 | Summer | Feathers | Floater | -19.08 | 13.34 | 3.37 |
| Striated Caracara | Franklin | 2014 | Summer | Feathers | Floater | -20.06 | 11.81 | 3.39 |
| Striated Caracara | Franklin | 2014 | Summer | Feathers | Floater | -19.52 | 13.87 | 3.33 |
| Striated Caracara | Franklin | 2014 | Summer | Feathers | Floater | -21.09 | 11.79 | 3.32 |
| Striated Caracara | Franklin | 2014 | Summer | Feathers | Floater | -21.34 | 11.84 | 3.37 |
| Striated Caracara | Franklin | 2014 | Summer | Feathers | Floater | -19.83 | 13.26 | 3.38 |
| Striated Caracara | Franklin | 2014 | Summer | Feathers | Floater | -18.08 | 13.96 | 3.32 |
| Striated Caracara | Franklin | 2016 | Summer | Feathers | Floater | -17.51 | 14.75 | 3.20 |
| Striated Caracara | Franklin | 2016 | Summer | Feathers | Floater | -19.83 | 13.14 | 3.40 |
| Striated Caracara | Franklin | 2016 | Summer | Feathers | Floater | -18.23 | 13.90 | 3.22 |
| Striated Caracara | Franklin | 2016 | Summer | Feathers | Floater | -18.96 | 13.03 | 3.23 |
| Striated Caracara | Franklin | 2016 | Summer | Feathers | Floater | -19.32 | 13.22 | 3.19 |
| Striated Caracara | Franklin | 2016 | Summer | Feathers | Floater | -18.94 | 13.31 | 3.20 |
| Striated Caracara | Franklin | 2016 | Summer | Feathers | Floater | -15.19 | 17.07 | 3.13 |
| Striated Caracara | Franklin | 2016 | Summer | Feathers | Floater | -19.26 | 13.72 | 3.43 |
| Striated Caracara | Franklin | 2016 | Summer | Feathers | Floater | -16.89 | 15.97 | 3.30 |
| Striated Caracara | Franklin | 2016 | Summer | Feathers | Floater | -17.27 | 15.02 | 3.23 |
| Striated Caracara | Franklin | 2016 | Summer | Feathers | Floater | -17.85 | 14.61 | 3.28 |
| Striated Caracara | Franklin | 2016 | Summer | Feathers | Floater | -19.26 | 13.61 | 3.38 |
| Striated Caracara | Franklin | 2016 | Summer | Feathers | Floater | -19.13 | 16.09 | 3.20 |
| Striated Caracara | Franklin | 2016 | Summer | Feathers | Floater | -20.00 | 12.62 | 3.23 |
| Striated Caracara | Franklin | 2016 | Summer | Feathers | Floater | -20.74 | 12.07 | 3.27 |
| Striated Caracara | Franklin | 2016 | Summer | Feathers | Floater | -18.95 | 12.94 | 3.23 |
| Striated Caracara | Franklin | 2016 | Summer | Feathers | Floater | -17.58 | 14.38 | 3.21 |
| Striated Caracara | Franklin | 2016 | Summer | Feathers | Floater | -16.98 | 14.49 | 3.13 |
| Striated Caracara | Franklin | 2016 | Summer | Feathers | Floater | -19.60 | 16.05 | 3.30 |
| Striated Caracara | Franklin | 2016 | Summer | Feathers | Floater | -14.29 | 16.65 | 3.21 |
| Striated Caracara | Franklin | 2016 | Summer | Feathers | Floater | -19.35 | 14.49 | 3.35 |
| Striated Caracara | Franklin | 2016 | Summer | Feathers | Floater | -17.89 | 16.28 | 3.26 |
| Striated Caracara | Franklin | 2016 | Summer | Feathers | Floater | -18.06 | 15.73 | 3.31 |
| Striated Caracara | Franklin | 2017 | Summer | Feathers | Floater | -19.53 | 13.74 | 3.24 |
| Striated Caracara | Franklin | 2017 | Summer | Feathers | Floater | -18.67 | 14.29 | 3.26 |
| Striated Caracara | Franklin | 2017 | Summer | Feathers | Floater | -18.56 | 13.17 | 3.16 |
| Striated Caracara | Franklin | 2017 | Summer | Feathers | Floater | -19.39 | 12.11 | 3.17 |
| Striated Caracara | Franklin | 2017 | Summer | Feathers | Floater | -18.40 | 14.00 | 3.20 |
| Striated Caracara | Franklin | 2017 | Summer | Feathers | Floater | -16.91 | 16.43 | 3.18 |
| Striated Caracara | Franklin | 2017 | Summer | Feathers | Floater | -20.10 | 12.42 | 3.25 |
| Striated Caracara | Franklin | 2017 | Summer | Feathers | Floater | -16.56 | 16.14 | 3.17 |
| Striated Caracara | Franklin | 2017 | Summer | Feathers | Floater | -17.68 | 15.33 | 3.20 |
| Striated Caracara | Franklin | 2017 | Summer | Feathers | Floater | -18.85 | 12.69 | 3.23 |
| Striated Caracara | Franklin | 2017 | Summer | Feathers | Floater | -18.72 | 13.82 | 3.24 |
| Striated Caracara | Franklin | 2017 | Summer | Feathers | Floater | -18.29 | 13.62 | 3.24 |
| Striated Caracara | Franklin | 2017 | Summer | Feathers | Floater | -17.53 | 14.98 | 3.31 |
| Striated Caracara | Franklin | 2017 | Summer | Feathers | Floater | -20.24 | 11.81 | 3.26 |
| Striated Caracara | Franklin | 2017 | Summer | Feathers | Floater | -16.84 | 14.99 | 3.24 |
| Striated Caracara | Franklin | 2017 | Summer | Feathers | Floater | -18.40 | 14.45 | 3.21 |
| Striated Caracara | Franklin | 2017 | Summer | Feathers | Floater | -18.63 | 13.84 | 3.22 |
| Striated Caracara | Franklin | 2017 | Summer | Feathers | Floater | -17.26 | 14.33 | 3.22 |
| Striated Caracara | Franklin | 2017 | Summer | Feathers | Floater | -20.28 | 12.48 | 3.37 |
| Striated Caracara | Franklin | 2017 | Summer | Feathers | Floater | -22.22 | 12.23 | 3.27 |
| Striated Caracara | Franklin | 2017 | Summer | Feathers | Floater | -17.55 | 15.15 | 3.27 |
| Striated Caracara | Franklin | 2017 | Summer | Feathers | Floater | -19.90 | 13.06 | 3.28 |
| Striated Caracara | Franklin | 2017 | Summer | Feathers | Floater | -20.61 | 11.75 | 3.30 |
| Striated Caracara | Franklin | 2017 | Summer | Feathers | Floater | -19.10 | 12.65 | 3.25 |
| Striated Caracara | Franklin | 2017 | Summer | Feathers | Floater | -19.34 | 13.00 | 3.28 |
| Striated Caracara | Franklin | 2017 | Summer | Feathers | Floater | -19.09 | 13.49 | 3.31 |
| Striated Caracara | Franklin | 2017 | Summer | Feathers | Floater | -20.18 | 12.05 | 3.30 |
| Striated Caracara | Franklin | 2017 | Summer | Feathers | Floater | -18.41 | 13.62 | 3.23 |
| Striated Caracara | Franklin | 2017 | Summer | Feathers | Floater | -19.10 | 12.62 | 3.22 |
| Striated Caracara | Franklin | 2014 | Summer | Blood | Chick | -20.84 | 11.45 | 3.25 |
| Striated Caracara | Franklin | 2014 | Summer | Blood | Chick | -21.76 | 10.15 | 3.40 |
| Striated Caracara | Franklin | 2014 | Summer | Blood | Chick | -21.41 | 10.03 | 3.25 |
| Striated Caracara | Franklin | 2014 | Summer | Blood | Chick | -21.70 | 10.47 | 3.29 |
| Striated Caracara | Franklin | 2014 | Summer | Blood | Chick | -20.52 | 11.12 | 3.28 |
| Striated Caracara | Franklin | 2014 | Summer | Blood | Chick | -18.04 | 14.93 | 3.32 |
| Striated Caracara | Franklin | 2014 | Summer | Blood | Chick | -21.48 | 10.34 | 3.28 |
| Striated Caracara | Franklin | 2014 | Summer | Blood | Chick | -21.58 | 10.31 | 3.35 |
| Striated Caracara | Franklin | 2014 | Summer | Blood | Chick | -21.03 | 10.34 | 3.31 |
| Striated Caracara | Franklin | 2014 | Summer | Blood | Chick | -22.10 | 10.54 | 3.42 |
| Striated Caracara | Franklin | 2014 | Summer | Blood | Chick | -21.90 | 10.48 | 3.35 |
| Striated Caracara | Franklin | 2014 | Summer | Blood | Chick | -21.72 | 10.32 | 3.32 |
| Striated Caracara | Franklin | 2014 | Summer | Blood | Chick | -21.56 | 10.32 | 3.31 |
| Striated Caracara | Franklin | 2016 | Summer | Blood | Chick | -22.12 | 9.83 | 3.26 |
| Striated Caracara | Franklin | 2016 | Summer | Blood | Chick | -21.32 | 10.08 | 3.23 |
| Striated Caracara | Franklin | 2016 | Summer | Blood | Chick | -21.85 | 9.95 | 3.27 |
| Striated Caracara | Franklin | 2016 | Summer | Blood | Chick | -21.63 | 9.78 | 3.33 |
| Striated Caracara | Franklin | 2016 | Summer | Blood | Chick | -20.52 | 11.13 | 3.26 |
| Striated Caracara | Franklin | 2016 | Summer | Blood | Chick | -20.42 | 11.07 | 3.26 |
| Striated Caracara | Franklin | 2016 | Summer | Blood | Chick | -21.23 | 9.60 | 3.39 |
| Striated Caracara | Franklin | 2016 | Summer | Blood | Chick | -21.57 | 10.01 | 3.31 |
| Striated Caracara | Franklin | 2016 | Summer | Blood | Chick | -20.85 | 11.20 | 3.22 |
| Striated Caracara | Franklin | 2016 | Summer | Blood | Chick | -20.89 | 10.96 | 3.40 |
| Striated Caracara | Franklin | 2016 | Summer | Blood | Chick | -21.55 | 10.05 | 3.24 |
| Striated Caracara | Franklin | 2016 | Summer | Blood | Chick | -21.51 | 9.62 | 3.34 |
| Striated Caracara | Franklin | 2017 | Summer | Blood | Chick | -19.96 | 11.21 | 3.26 |
| Striated Caracara | Franklin | 2017 | Summer | Blood | Chick | -19.99 | 11.66 | 3.31 |
| Striated Caracara | Franklin | 2017 | Summer | Blood | Chick | -20.35 | 11.80 | 3.28 |
| Striated Caracara | Franklin | 2017 | Summer | Blood | Chick | -20.58 | 11.54 | 3.40 |
| Striated Caracara | Franklin | 2017 | Summer | Blood | Chick | -20.16 | 12.19 | 3.29 |
| Striated Caracara | Franklin | 2017 | Summer | Blood | Chick | -20.09 | 12.06 | 3.28 |
| Striated Caracara | Franklin | 2017 | Summer | Blood | Chick | -20.33 | 11.84 | 3.42 |
| Striated Caracara | Franklin | 2017 | Summer | Blood | Chick | -20.20 | 11.37 | 3.37 |
| Striated Caracara | Franklin | 2017 | Summer | Blood | Chick | -20.30 | 11.34 | 3.26 |
| Striated Caracara | Franklin | 2017 | Summer | Blood | Chick | -19.30 | 12.28 | 3.45 |
| Striated Caracara | Franklin | 2017 | Summer | Blood | Chick | -19.50 | 12.32 | 3.29 |
| Striated Caracara | Franklin | 2017 | Summer | Blood | Chick | -19.69 | 11.86 | 3.27 |
| Striated Caracara | Franklin | 2017 | Summer | Blood | Chick | -19.63 | 11.69 | 3.30 |
| Striated Caracara | Franklin | 2017 | Summer | Blood | Chick | -18.94 | 12.16 | 3.24 |
| Striated Caracara | Franklin | 2017 | Summer | Blood | Chick | -20.26 | 11.97 | 3.30 |
| Striated Caracara | Franklin | 2017 | Summer | Blood | Chick | -20.08 | 11.20 | 3.33 |
| Striated Caracara | Franklin | 2017 | Summer | Blood | Chick | -19.27 | 12.28 | 3.34 |
| Striated Caracara | Franklin | 2017 | Summer | Blood | Chick | -18.88 | 12.43 | 3.25 |
| Striated Caracara | Franklin | 2016 | Summer | Feathers | Breeding adult | -20.06 | 12.75 | 3.20 |
| Striated Caracara | Franklin | 2016 | Summer | Feathers | Breeding adult | -19.73 | 12.10 | 3.20 |
| Striated Caracara | Franklin | 2016 | Summer | Feathers | Breeding adult | -20.07 | 12.69 | 3.25 |
| Striated Caracara | Franklin | 2016 | Summer | Feathers | Breeding adult | -19.13 | 13.41 | 3.24 |
| Striated Caracara | Franklin | 2017 | Summer | Feathers | Breeding adult | -18.03 | 14.40 | 3.17 |
| Striated Caracara | Franklin | 2017 | Summer | Feathers | Breeding adult | -19.76 | 12.63 | 3.19 |
| Striated Caracara | Franklin | 2017 | Summer | Feathers | Breeding adult | -15.87 | 17.55 | 3.23 |
| Striated Caracara | Franklin | 2017 | Summer | Feathers | Breeding adult | -20.51 | 11.99 | 3.18 |
| Striated Caracara | Franklin | 2017 | Summer | Feathers | Breeding adult | -20.52 | 12.60 | 3.21 |
| Striated Caracara | Franklin | 2017 | Summer | Feathers | Breeding adult | -19.79 | 13.03 | 3.16 |
| Striated Caracara | Franklin | 2017 | Summer | Feathers | Breeding adult | -19.60 | 12.75 | 3.19 |
| Striated Caracara | Franklin | 2017 | Summer | Feathers | Breeding adult | -19.25 | 13.60 | 3.20 |
| Striated Caracara | Franklin | 2017 | Summer | Feathers | Breeding adult | -19.02 | 13.76 | 3.19 |
| Sea Lion | Observatorio | 2017 | Winter | Faeces | Adult | -20.15 | 15.00 | 3.92 |
| Sea Lion | Observatorio | 2017 | Winter | Faeces | Adult | -16.36 | 16.30 | 3.65 |
| Sea Lion | Observatorio | 2017 | Winter | Faeces | Adult | -19.80 | 14.87 | 3.82 |
| Red deer | Franklin | 2018 | Summer | Muscle | Adult | -24.78 | 3.01 | 2.66 |
| Red deer | Franklin | 2018 | Summer | Muscle | Adult | -25.02 | 9.52 | 2.82 |
| Red deer | Franklin | 2018 | Summer | Muscle | Adult | -24.99 | 1.19 | 2.71 |
| Feral goat | Franklin | 2018 | Summer | Muscle | Adult | -24.66 | 3.34 | 2.82 |
| Upland goose | Franklin | 2018 | Summer | Muscle | Adult | -27.15 | 11.48 | 3.00 |
| Upland goose | Franklin | 2018 | Summer | Muscle | Chick | -33.66 | 12.93 | 4.73 |
| Insect | Franklin | 2018 | Summer | Muscle | Adult | -27.50 | 2.59 | 4.32 |
| Insect | Franklin | 2018 | Summer | Muscle | Adult | -27.73 | 3.40 | 4.26 |
| Insect | Franklin | 2018 | Summer | Muscle | Adult | -28.55 | 8.54 | 4.44 |
| Insect | Franklin | 2018 | Summer | Muscle | Adult | -26.63 | 15.20 | 3.99 |
| Insect | Franklin | 2018 | Summer | Muscle | Adult | -28.14 | 2.06 | 4.04 |
| Insect | Franklin | 2018 | Summer | Muscle | Adult | -28.93 | 2.61 | 4.22 |
| Chuanisín mouse | Franklin | 2018 | Summer | Muscle | Adult | -21.32 | 7.05 | 3.36 |
| Chuanisín mouse | Franklin | 2018 | Summer | Muscle | Adult | -25.02 | 2.42 | 3.20 |
| Chuanisín mouse | Franklin | 2018 | Summer | Muscle | Adult | -25.25 | 4.69 | 3.18 |
| Rat | Franklin | 2018 | Summer | Muscle | Adult | -15.09 | 14.92 | 3.08 |
| Rat | Franklin | 2018 | Summer | Muscle | Adult | -21.31 | 6.44 | 3.59 |
| Rat | Franklin | 2018 | Summer | Muscle | Adult | -23.07 | 7.23 | 3.71 |
| Rockhopper penguin | Franklin | 2018 | Summer | Muscle | Chick | -18.70 | 13.31 | 3.37 |
| Rockhopper penguin | Franklin | 2018 | Summer | Muscle | Chick | -20.36 | 12.72 | 3.18 |
| Rockhopper penguin | Franklin | 2018 | Summer | Muscle | Chick | -20.40 | 10.42 | 3.41 |
| Rockhopper penguin | Franklin | 2018 | Summer | Muscle | Chick | -21.16 | 10.39 | 3.26 |
| Rockhopper penguin | Franklin | 2018 | Summer | Muscle | Chick | -22.01 | 10.25 | 3.12 |
| Rockhopper penguin | Franklin | 2018 | Summer | Muscle | Chick | -22.14 | 10.24 | 3.60 |
| Rockhopper penguin | Franklin | 2018 | Summer | Muscle | Chick | -21.70 | 9.90 | 3.50 |
| Rockhopper penguin | Franklin | 2018 | Summer | Muscle | Chick | -21.27 | 9.38 | 3.16 |
| Rockhopper penguin | Franklin | 2018 | Summer | Muscle | Chick | -23.42 | 9.00 | 4.11 |
| Rockhopper penguin | Franklin | 2018 | Summer | Muscle | Chick | -23.05 | 8.66 | 3.84 |
| Rockhopper penguin | Franklin | 2018 | Summer | Muscle | Chick | -22.35 | 8.65 | 3.56 |
| Rockhopper penguin | Franklin | 2018 | Summer | Muscle | Adult | -22.68 | 8.10 | 3.44 |
| Rockhopper penguin | Franklin | 2018 | Summer | Muscle | Adult | -23.15 | 7.87 | 3.19 |
| Rockhopper penguin | Franklin | 2018 | Summer | Muscle | Egg | -20.89 | 9.96 | 3.19 |
| Rockhopper penguin | Franklin | 2018 | Summer | Muscle | Egg | -21.61 | 9.40 | 3.26 |
| Rockhopper penguin | Franklin | 2018 | Summer | Muscle | Egg | -21.86 | 9.29 | 3.26 |
| Imperial Shag | Franklin | 2018 | Summer | Muscle | Chick | -16.49 | 15.82 | 3.32 |
| Imperial Shag | Franklin | 2018 | Summer | Muscle | Chick | -17.21 | 15.44 | 3.99 |
| Imperial Shag | Franklin | 2018 | Summer | Muscle | Chick | -16.97 | 15.23 | 3.47 |
| Imperial Shag | Franklin | 2018 | Summer | Muscle | Chick | -16.36 | 14.78 | 3.20 |
| Imperial Shag | Franklin | 2018 | Summer | Muscle | Chick | -16.33 | 14.04 | 3.16 |
| Imperial Shag | Franklin | 2018 | Summer | Muscle | Chick | -17.19 | 13.21 | 3.32 |
| Imperial Shag | Franklin | 2018 | Summer | Muscle | Egg | -14.80 | 16.34 | 3.09 |
| Imperial Shag | Franklin | 2018 | Summer | Muscle | Egg | -15.03 | 15.79 | 3.12 |
| Imperial Shag | Franklin | 2018 | Summer | Muscle | Egg | -15.61 | 14.88 | 3.12 |
| Mussel | Franklin | 2017 | Summer | Muscle | Adult | -15.09 | 10.21 | 3.18 |
| Mussel | Franklin | 2017 | Summer | Muscle | Adult | -14.33 | 11.32 | 3.20 |
| Mussel | Franklin | 2017 | Summer | Muscle | Adult | -14.64 | 11.52 | 3.17 |
| Mussel | Franklin | 2017 | Summer | Muscle | Adult | -14.54 | 10.56 | 3.32 |
| Mussel | Franklin | 2017 | Summer | Muscle | Adult | -14.74 | 10.60 | 3.10 |
